# Survey of Risk Factors and Genetic Characterization of Ewe Neck in a World Population of Pura Raza Español Horses

**DOI:** 10.3390/ani10101789

**Published:** 2020-10-01

**Authors:** María Ripolles, María J. Sánchez-Guerrero, Davinia I. Perdomo-González, Pedro Azor, Mercedes Valera

**Affiliations:** 1 Department of Agro-Forestry Sciences, ETSIA, University of Seville, Carretera de Utrera Km 1, 41013 Sevilla, Spain; mripolles@alumni.unav.es (M.R.); daviniapergon@gmail.com (D.I.P.-G.); pedroazor@lgancce.com (P.A.); mvalera@us.es (M.V.); 2 Department of Molecular Biology and Biochemistry Engineering, Universidad Pablo de Olavide, Carretera de Utrera Km 1, 41013 Sevilla, Spain

**Keywords:** heritability, morphological defect, Spanish purebred horses, upside-down neck

## Abstract

**Simple Summary:**

Ewe Neck is a common morphological defect of the Pura Raza Español (PRE) population, which seriously affects the horse’s development. In this PRE population (35,267 PRE), a total of 9693 animals (27.12% of total) was Ewe Neck-affected. It has been demonstrated that genetic and risk factors (sex, age, geographical area, coat color, and stud size) are involved, being more prevalent in the males, 4–7 years old, chestnut coat, from small studs (less than 5 mares), and raised in North America. The morphological traits height at chest, length of back, head-neck junction, and bottom neck-body junction and the body indices, head index, and thoracic index were those most closely related with the appearance of this morphological defect. The additional genetic base of Ewe Neck in PRE, which presents low-moderate heritability (h2: 0.23–0.34), shows that the prevalence of this defect could be effectively reduced by genetic selection.

**Abstract:**

Ewe Neck is a relatively common morphological defect in Pura Raza Español (PRE) horses and other Baroque type horse breeds, which adversely affects the breeding industry; (1) objectives: to establish the within-breed prevalence, possible associated factors, and heritability of Ewe Neck in PRE horses; (2) methods: the database included evaluations of 35,267 PRE horses. The Ewe Neck defect, 16 morphological traits, and 4 body indices were recorded. A Bayesian genetic animal model included the following systematic effects: sex, age, coat color, geographical area of the stud, and birth stud size were used; (3) results: in this PRE population, a total of 27.12% was affected. All the risk factors studied were significantly associated with the Ewe Neck score. The heritability coefficient for Ewe Neck score ranged from 0.23 to 0.34. Morphological traits (height at chest, length of back, head-neck junction, and bottom neck-body junction) and the indices (head and thoracic index) were those most closely related with the appearance of Ewe Neck; (4) conclusions: Ewe Neck is a relatively frequent defect in PRE horses, associated with risk factors and other morphological traits, with a moderate level of heritability. Breeding to select against this condition may therefore be beneficial in this breed.

## 1. Introduction

The Pura Raza Español (PRE) horse has its origin in Baroque horses, as do other equine breeds such as Friesian, Lipizzaner, Frederiksberg, Lusitano, and Kaldruby [1,2,3], although the PRE horse is the most representative breed in the Baroque group. It is the oldest equine breed in Spain, with a studbook created in 1912. The breed census currently stands at 252,852 animals (125,824 stallions and 127,010 mares) [4]. Since the 1960s, breeding animals have been regularly exported to other countries to establish local subpopulations, which are now distributed in more than 66 countries, with 23.29% of registered horses bred in foreign countries [5]. The popularity of this breed lies in its inherent ethological and morphological attributes and its excellent sports skills in certain disciplines, such as dressage [6,7]. The PRE is characterized by harmonious conformation, a subconvex front profile, with a docile and energetic temperament. PRE have an average wither height between 158 and 161.5 cm; the neck on average measures 73–76 cm [8], and is muscular, inserted into the body above the point of shoulder, with a fine upper edge, curving upwards from the withers to the head. The neck is inserted deep into the trunk, but this is less so at the head. From 2003, the breed was subjected to a breeding program, managed by the National Purebred Spanish Horse Breeders’ Association (ANCCE), which includes functional conformation, riding, and dressage ability as its main selection criteria [6,7].

The corporal regions in horses are especially important in movement and sport performance [9,10], and there are morphologic selection indices, where the morphological traits are chosen according to conformation and movement for dressage [7]. Head and neck positions are two crucial parameters in the conformation of sport horses [11]. In numerous studies, the influence of the position of the head and neck has been analyzed [12,13], and their relationship with locomotion and various pathological factors that may affect these regions has been studied [14,15,16].

Conformational traits and morphological defects affect equine health and the sale price of the horses [17,18]. The identification of genetic diseases has been hampered in horses due to their long gestation, single births, dispersion of horses after weaning, and the existence of many diseases with a delayed onset or variable severity [19]. Despite the number of inherited disorders affecting horses [20,21], there has been little research on specific inherited disorders, with a shortage of work addressing the genetic study of the disease or the defect from a population point of view. Some studies estimate the heritability of musculoskeletal defects or diseases as osteochondrosis dissecans in Maremmano horses [22], Hanoverian warmblood horses [23], and Swiss Warmblood horses [24]; suspensory ligament injury and tendon injury in Thoroughbred racehorses [17]; osteoarthrosis in distal and proximal interphalangeal joints, fetlock, hock, and stifle joints in Hanoverian warmblood [25]; tarsocrural osteochondrosis and palmar first phalanx osteochondral fragments in Standardbred trotters [26]; the deformity of the dorsal edge of the neck (Cresty Neck) in PRE horses [27]; and Club Foot in Arabian Pureblood horses [28]. Further characterization of the equine genome and identification of mutations in genes associated with muscular disorders, has led to important advances in the field of inherited skeletal muscle disease research [29].

In the Baroque equine breeds and specifically in PRE horses, the main defects related to the horse’s neck are Cresty Neck and Ewe Neck. Ewe Neck (also known as Swan Neck, Turkey Neck, and Upside-down Neck) is one of the disqualifying morphological defects in PRE horses. So, PRE breeders’ associations has included a score of four for Ewe Neck as a disqualifying defect in their assessment of basic breeding aptitude, which implies that a horse with a high degree of Ewe Neck cannot be registered in the main section of the herd book nor leave offspring in the breed. Despite this, the defect continues to appear and horses with a phenotypic presentation less than a scale of three are used as stallions. Surprisingly, there is no previous study that addresses the Ewe Neck prevalence, the possible risk factors or the genetic of this defect in any equine breed. So, the physiological causes of the morphological defect of the Ewe Neck are currently unknown. It seems to be multifactorial in nature but may relate to hyper-musculation of the ventral neck region (sternocephalic and brachiocephalic muscle) [30] and bad practices carried out during equestrian sports [12].

It is crucial to understand better how different factors contribute to the development of Ewe Neck in order to improve both the management and breeding selection of PRE horses. The specific aims of this work were to:

(1) Calculate the within-breed prevalence of Ewe Neck in a significant population of PRE horses.
(2) Determine whether the development of Ewe Neck in this breed is associated with age, sex, coat color, or stud geographical location and size, all of which are closely related with management.
(3) Estimate the heritability for Ewe Neck and the genetic correlations with certain morphological traits and body indices to incorporate the Ewe Neck trait into the PRE improvement program to lower the prevalence in the breed or even so that its incidence in the population disappears.

## 2. Materials and Methods

### 2.1. Description of Traits and Database

The database included records of 35,267 animals (23,090 mares and 12,177 stallions) with a mean age of 5.02 (1.99 S.D.) years old. These records were taken between 2012 and 2018, from 6 different geographical areas (Spain, rest of Europe, North America, Central America, South America, and Australia), during the morphological evaluation that all PRE horses have once in their life before being registered in the main section of the PRE herd book [27]. The evaluations were carried out by 18 previously trained veterinarians who travel around the world assessing horses for registration in the studbook. The following morphological traits were evaluated on a horse-by-horse basis (Table 1).

All the morphological measurements were taken from the left side of the horse while it was standing on a hard surface and flat ground, assuming a natural position. The horses were positioned for measurement with the front legs and hind feet parallel and as near to perpendicular as possible; the toes were in line. No sedatives were used. The instruments used for this type of evaluation were a flexible tape measure, for perimeter measurements and a zoometric cane for measuring elevations, distances, and widths.

**Figure 1:**
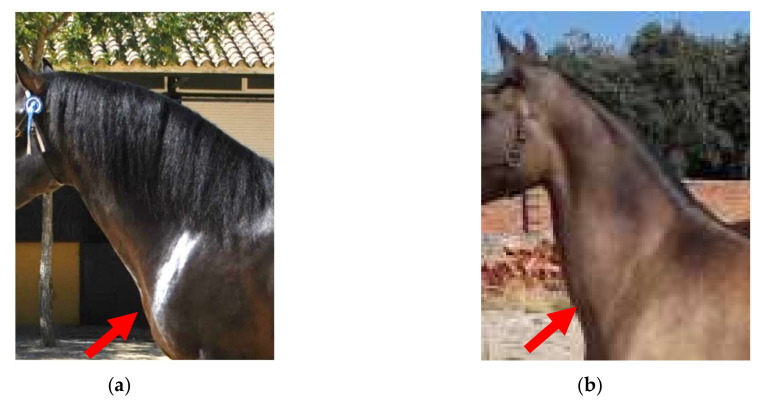
Ewe Neck in Pura Raza Español horse: (**a**) the morphological defect not present and (**b**) morphological defect Ewe Neck.

To carry out this study, the Ewe Neck trait was analyzed on a lineal scale, which includes 4 scores in which the extremes represent the biological limits for this morphological trait (Table 2, Figure 1).

All the analyses were performed three times after dividing the database into two datasets that are (i) whole population (*n* = 35,267) and (ii) affected Ewe Neck subpopulation (*n* = 9693):

1. Approach A—whole population with Ewe Neck score as a scale (*n* = 35,267). Ewe Neck score as a scale (classes 0, 1, 2, and 3).
2. Approach B—whole population with Ewe Neck score as a dichotomic trait (*n* = 35,267). Ewe Neck score as a dichotomic trait (0 (no affected, class 0) and 1 (affected, classes 1, 2, and 3)).
3. Approach C—Affected Ewe Neck subpopulation (classes 1, 2, and 3) (*n* = 9693).

The potential risk factors studied were:

- Sex (2 levels): male and female.
- Age (3 levels): 1 to 4 years, 4 to 7 years, and more than 7 years.
- Coat color (4 levels): grey, bay, black, and chestnut.
- Geographical area (6 levels): Spain, rest of Europe, North America, Central America, South America, and Australia.
- Birth stud size (5 levels): less than 5 mares, 5 to 9 mares, 10 to 19 mares, 20 to 50 mares, and more than 50 mares.

### 2.2. Descriptive Statistic and Risk Analysis

The number of PRE horses, in each Ewe Neck class, affected by each risk factor was present in Table 2. A multivariate Generalized Non-linear Model (GLZ) was used to examine associations between the morphological traits with the potential risk factors. An association frequency analysis of all the risk factors (Maximum-Likelihood Chi-square) was also carried out. Statistical analyses were performed using Statistica 11 for Windows software [31].

### 2.3. Genetic Model

Genetic parameters of Ewe Neck were estimated by three complementary approaches:

- Whole population (1) (*n* = 35,267; 4 levels of Ewe Neck: class 0, 1, 2 and 3). Approach A.
- Dichotomy of whole population (2) (*n* = 35,267; 2 levels of Ewe Neck: level 1 animals without neck defect, class 0 and level 2 which includes class 1, 2, and 3 of the Ewe Neck defect). Approach B.
- Affected Ewe Neck subpopulation (*n* = 9565; 3 levels of Ewe Neck: class 1, 2, 3). Approach C.

Genetic parameters between Ewe Neck and the other morphological traits were estimated using best linear unbiased prediction (BLUP) evaluation based on a bivariate animal linear model with Threshold model (TM) software [32], using a Bayesian approach. The equation in matrix notation to solve the mixed model was:

(1) y=Xb+Zu+e,

with:

(2) $$\left(\begin{array}{c} u\\ e \end{array}\right)\sim N\left(\left[\begin{array}{c} 0\\ 0 \end{array}\right],\;\left[\begin{array}{cc} A\sigma \;u\;2\; & 0\\ 0 & I\sigma \;e\;2\; \end{array}\right]\right),$$

where y is the vector of observations, X is the incidence matrix of systematic effects, Z is the incidence matrix of animal genetic effects, b is the vector of systematic effects, u is the vector of direct animal genetic effects, **e** is the vector of residuals, σ _u_^2^ is the direct genetic variance, σ _e_^2^ is the residual variance, I is an identity matrix, and A is the numerator relationship matrix.

The fitted model included the following systematic effects for each specific trait being analyzed: age as a linear covariable; sex (2 levels); the stud geographical area (6 levels); the coat color (4 levels); and birth stud size (5 levels). Marginal posterior distributions of all parameters were estimated using the Gibbs sampling algorithm [33]. Total Gibbs chain lengths of 1,000,000 samples for each analysis were defined, with a burn-in period of 100,000 and a thinning interval of 100.

The pedigree file information necessary for genetic evaluation, collected from the official PRE studbook, included a minimum of 4 generations (82,488 horses).

## 3. Results

A total of 35,267 PRE horses were evaluated; 25,702 horses (72.88%) did not have Ewe Neck (class 0); 5339 horses (15.14%) had an incipient Ewe Neck (class 1); 3989 horses (11.31%) had a noticeable appearance of Ewe Neck (class 2) and a total of 237 horses (0.67%) had a very serious Ewe Neck defect (class 3) (Table 3).

The risk factors associated with Ewe Neck score are shown in Table 4. In the whole population, all the risk factors studied (sex, age, coat color, geographical area, and livestock size) were significant risk factors, with significance coefficients below 0.05. Stallions had a significantly higher frequency Ewe Neck score than females in the whole population. Horses between 4–7 years (28.40% Ewe Neck horses) had a higher Ewe Neck score than horses over 7 years old (27.95%) and under 4 years old (25.05%) in the whole population.

Horses with black and chestnut coats had higher percentages of horses affected by Ewe Neck than horses with brown and grey coat colors. The biggest significant difference was between horses with grey coats (25.26% Ewe Neck horses) and horses with chestnut coats (32.37%).

There was also a significant association with the Ewe Neck score and the country in which the horse was located. In general, the percentage of Ewe Neck-affected horses was lower in studs located in Europe and Spain than in other geographical areas. The biggest significant difference was between the percentage of horses with Ewe Neck in Europe (22.96% Ewe Neck horses) and the North American countries group (32.03%).

There was also a significant association with the Ewe Neck score and the stud size (according to the number of mares). In general, Ewe Neck-affected horses were lower in studs where there were between 20–50 mares than studs with less than 20 mares. The biggest significant percentages of animals with Ewe Neck defect was in stud with 20–50 mares (25.87% Ewe Neck horses) while stud with less than 5 mares had a percentage of 29.45% PRE horses affected with Ewe Neck.

The genetic parameters (heritability and genetic correlations) in the three complementary approaches (A, B, and C) between morphological traits and horses affected by Ewe Neck are shown in Table 5.

Ewe Neck heritability ranged from 0.23 in Approach A (whole population scale) to 0.34 in Approach C (only population affected). The estimated heritability for the morphological traits and body indices oscillated between medium and high values (0.26 for head-neck junction and 0.77 for height at withers).

In general, most of the genetic correlations between the Ewe Neck score and morphological traits were negative with maximum of −0.38 (width of chest in horses affected by Ewe Neck). Few genetic correlations were positive: width of chest, dorso-sternum diameter, length of loin, length of back, and body index, with a maximum value of 0.57 (head-neck junction in horses affected by Ewe Neck—Approach *C*). The genetic correlations showed low to medium values (0.00 to 0.57 for width of chest and Ewe Neck with Approach A and head-neck junction with Approach C).

## 4. Discussion

This study has addressed for the first time the within-breed prevalence of Ewe Neck within a large worldwide population of PRE horses and examined the risk factors for this condition in this breed. Ewe Neck is a prevalent morphological defect in this breed and nearly 27.12% of this population has this defect, ranging in severity from a noticeable Ewe Neck (Class 1) (15.14%) to serious Ewe Neck (Class 3) (0.67%). There are no previous data of within-breed prevalence of Ewe Neck scores in horses and there may be an underrepresentation of horses affected by Ewe Neck, due to the fact that some horses presenting a high level of the defect cannot be brought to be valued, have been slaughtered, or have not survived.

Curiously, PRE stallions have a higher prevalence of Ewe Neck than mares (38.18% for stallions and 26.55% for mares); this also occurs with Cresty Neck [27]. The PRE stallion’s neck is usually more voluminous, and stallions are used for sport more often than PRE mares. Another cervical disease, stenotic myelopathy, has been studied previously and its prevalence was also higher in stallions than in females [34]. Age also was a relevant risk factor for Ewe Neck, with young horses (4–7 years old) showing higher percentages of those affected. This could indicate that the Ewe Neck is evident at an early age, which does not happen in the case of Cresty Neck [34]. In fact, the morphological defect Ewe Neck is a birth defect; however, it is possible that age as a risk factor will be more evident due to external factors, as it is clearly evident at four years old. So, we hypothesize that at later ages, studs do not present PRE horses with Ewe Neck because they have already been discarded.

A higher percentage of horses with black and chestnut coat colors were affected by Ewe Neck. This may be because, for a long time, these animals have been chosen as stallions or mares for this coat colour and not for their morphology (even despite knowing they had this defect). In fact, black stallions are chosen as breeding animals at very early ages [35]. Horses with chestnut coat color were forbidden in the PRE population until 2002, so the selection intensity in this subpopulation was probably lower compared with the other coats [8]. The association of coat color and hereditary diseases can be explained by the concept of pleiotropy [36], as in the case of horses with grey coat color, whose mutations are involved in the development of melanocytes and the emergence of genetic diseases [36]. Coat color also is a significant risk factor for Cresty Neck, vitiligo, and melanoma in PRE [27,37]. However, further studies would be necessary to determine if pleiotropy exists between the genes that determine the coat color and the Ewe Neck.

We did not come up with a definitive explanation for the influence of the geographic location of the stud where horses were resident on the Ewe Neck score, but it may be related to differences in horse management. For example, a slight Ewe Neck could be positively influenced by the development of the dorsal neck muscles due to training; it is a hypothesis which would positively affect to trained horses. It is also possible that moderate differences in the genetic pool of subpopulations of PRE horses in different parts of the world (since they could originate from the founders which carried this defect) [5] may have contributed to the differences observed in the prevalence of Ewe Neck. This also happened in the case of Cresty Neck, vitiligo, and melanoma, where, curiously, the same geographical regions (American region) show higher prevalence of PRE affected [27,37].

Another possibility is that depending on the geographical location of the stud, Ewe Neck may be related to differences in horse. The use and the management of the horse are linked to the neck characteristics. According to Lesimple [38], horses that were ridden less frequently, had opportunities to graze, and were living in groups, showed more cervical flexion (a more rounded topline of the neck) at quiet stance than horses that were stabled, ridden daily, and kept without grazing. The management of the horse could also be related with the stud size. In large studs, the most selection is made by the farmer and therefore the defect will be less prevalent (25.87% of Ewe Neck affected horses in studs with 20–50 mares and 29.45% in stud with less than 5 mares). In these large studs, the breeder has the option of choosing which horses will be breeders (those which have no morphological defects) and they presumably want to sell the foals at the best possible price, so, theoretically, defects must occur less frequently.

From a genetic point of view, we have less information on the heritability of morphological disorders in horses compared to other important animal species, such as in cows [39,40,41,42], mainly due to the relatively high cost and time involved in researching inheritance patterns in horses [27]. Closed Genealogical Books, the selective breeding system, and repeated use of most popular stallions are associated with an increase in inherited disorders in horses.

There are few studies about the origin of the defect, although the evidence suggests it has a hereditary character, linked to the existence of a significant number of genes. The hereditary character of most of morphological defect has motivated breeders’ associations to include it as a disqualifying defect in their assessment of basic breeding aptitude; however, its multifactorial nature and recessive behavior mean that sacrificing these animals is not a sufficiently reliable way of fighting against the disease, as the carriers are not detected.

The heritability estimated for Ewe Neck was low-medium, in the same range as that estimated in other morphological defects, such as Cresty Neck (0.37) by our group [27]. The heritability obtained for morphological traits was similar to the results previously obtained [8,43,44,45] in PRE horses by our group being high in heights (about 0.80) and lower in linear traits and angles (about 0.30), and in other horse breeds with similar traits, in Warmblood horses ranging from 0.02 to 0.77 [46], and in Swedish Warmblood horses [47] ranging from 0.24 to 0.58. A horse’s size and body conformation are presumably subject to a strict process of selection over time [48], which has led to PRE stallions and mares being currently significantly taller and longer [45]. The most relevant morphological traits affected or not by Ewe Neck were height at chest, length of back, head-neck junction, and bottom neck-body junction, and the body indices (head index and thoracic index). These all had a negative genetic correlation, so the taller a PRE horse was at height at chest and the longer back it had, the less the possibility of having Ewe Neck. In addition, a closely packed bottom neck-body junction and a very indistinct head-neck junction help to avoid Ewe Neck. In the body indices case, it seems that proportionally wider animals have lesser Ewe Neck scores. If we analyze these correlations within the animals affected (approach C), the only relevant relationships were length of shoulder, length of back and loin, and head-neck junction (positive correlation) and dorso-sternum diameter (negative correlation). The majority of these traits have previously been related with PRE functionality [7,45], but unfortunately we have not found previous studies that address the genetic correlations of Ewe Neck with other morphological traits.

Regarding the approach used, we suggest that in the PRE genetic evaluations, the Approach A should be used since it can determine at what level the defect occurs and it uses the entire evaluated population. This approach, allow a better kinship relationships in the matrix are more consistent and the genetic breeding value of each animal can be estimated, including which ones present the defect but are capable of transmitting it. It is necessary to carry out in the PRE horse, as in other Baroque equine breeds that present the Ewe Neck defect, a selection of horses against this defect since it provides an economic benefit in the breed both for improving the conformation of the horse and for improving it from a functional point of view.

## 5. Conclusions

Ewe Neck is a common morphological defect that affects 27.12% of the PRE population evaluated. Genetic and risk factors (sex, age, geographical area, coat color, and stud size) are involved in its development, being more prevalent in the males, 4–7 years old, chestnut coat, from small studs (less than 5 mares), and raised in North America. However, many horses presenting a high level of Ewe Neck have not been analyzed, as they have either been slaughtered or have not survived; therefore, the prevalence of the defect could in fact be much higher than the figure found in this study.

Genetic base of Ewe Neck in PRE, which presents low-moderate heritability, shows that the prevalence of this defect could be reduced by genetic selection using the Ewe Neck breeding values in all the PRE horses as a selection criterion into its breeding program. The additional morphological traits height at chest, length of back, head-neck junction, and bottom neck-body junction, and the body indices (head index and thoracic index) were those most genetically closely related with this morphological defect.

## Figures and Tables

**Table 1:** Description of the 16 morphological traits and 5 body indices analyzed in the Pura Raza Español Horse.

Morphological Traits		Description
**Morphological measurement (cm)**	Height at withers (HW)	Distance between the ground and the highest point of the withers.
	Height at decline point of withers (HDPW)	Distance between the ground and the lowest point of the withers.
	Height at chest (HC)	Distance between the ground and the hollow substernal.
	Width of head (WH)	Distance between the most protruding edge of the zygomatic arches.
	Length of neck (LN)	Distance between the base of the ear and the middle point of the spine of the scapula.
	Length of shoulder (LS)	Distance between the withers and the greater tubercle of the humerus (caudal part).
	Width of chest (WC)	Distance measured between cranio-lateral points of the humerus in the scapular-humeral articulations (left and right).
	Scapulo-isquial length (SIL)	Distance between the greater tubercle of the humerus (caudal part) and ischial tuberosity.
	Length of back (LBa)	Distance from the withers to the last thoracic vertebra.
	Length of loin (LL)	Distance between the last thoracic vertebra and the tuber coxae of the ilium.
	Dorso-sternum diameter (DSD)	Distance measured from the lowest point in the withers decline to the sternal area.
	Perimeter of thorax (PT)	Perimeter of thorax, measured at its midpoint.
**Linear conformation traits (class)**	Angle of shoulder (AS)	Angle formed by the line from the withers to the shoulder with the horizontal.
	Muscle development (MD)	Degree of body musculature at the level of back, loin, rump and thigh, ranging from very thin (class 1) to very muscular (class 9).
	Head-neck junction (HNJ)	Type of insertion of the head in the neck ranging from very distinct (class 1) to very indistinct (class 9).
	Bottom neck-body junction (NBJ)	Distance between the point of insertion neck-body (the ventral part) and the line connecting the 2 shoulder joints: scapula/humeral; ranging from very packed (class 1) to very marked (class 9).
**Body Indices**	Proportionality index (PrI)	[Height at withers/Scapulo-isquial length] × 100
	Head index (HI)	[Width of head/Length of head] × 100
	Thoracic index (TI)	[Bicostal Diameter/Dorso-Sternum Diameter] × 100
	Body index (BI)	[Height at withers/Perimeter of thorax] × 100

**Table 2:** Description of Ewe Neck scoring system in the Pura Raza Español Horse.

Score	Description
0	Absent Ewe Neck (Figure 1a).
1	Incipient Ewe Neck: ventral neck region (sternocephalic and brachiocephalic muscle) are slightly more developed than dorsal neck region (the Interscapular and adjacent prescapular). The horse does not have great difficulties of movement.
2	Noticeable appearance of Ewe Neck: The musculature of the ventral neck region is more developed than the dorsal neck region. The lower edge of the neck is slightly convex. The horse has difficulty making some movements.
3	Very serious Ewe Neck: hyper-musculature of the ventral neck region. The upper edge of the neck is slightly concave, and the lower edge is convex (deer neck). The horse has difficulties of movement (Figure 1b).

**Table 3:** Number of Pura Raza Español horses according to its Ewe Neck score class, depending on different risk factors.

Risk Factors		Number of Horses			
		Class 0	Class 1	Class 2	Class 3
Sex	Male (*n* = 12,177)	8745	1931	1409	92
	Female (*n* = 23,090)	16,957	3408	2580	145
Age	<4 years (*n* = 12,773)	9573	1843	1296	61
	4–7 years (*n* = 17,424)	12,476	2766	2052	130
	>7 years (*n* = 5070)	3653	730	641	46
Coat Color	Grey (*n* = 15,636)	11,756	2172	1624	84
	Brown (*n* = 13,006)	9396	2047	1469	94
	Black (*n* = 4438)	3071	693	639	35
	Chestnut (*n* = 2187)	1479	427	257	24
Geographical area	Spain (*n* = 26,482)	19,459	4078	2814	131
	North America (*n* = 3013)	2048	485	441	39
	Central America (*n* = 2726)	1928	423	360	15
	Europe (*n* = 1578)	1216	170	173	19
	South America (*n* = 1376)	983	169	194	30
	Australia (*n* = 92)	68	14	7	3
Birth stud size	<5 (*n* = 4054)	2860	621	525	48
	5–9 (*n* = 3821)	2725	566	501	29
	10–19 (*n* = 5742)	4153	843	701	45
	20–50 (*n* = 9701)	7191	1382	1071	57
	>50 (*n* = 11,949)	8773	1927	1191	58
**Total**		**25,702**	**5339**	**3989**	**237**

*n* = number of horses.

**Table 4:** Generalized Non-linear Model (GLZ) between the morphological traits with the risk factors and the percentage of subpopulation affected by Ewe Neck (horses with classes 1, 2, and 3), with an association frequency analysis of risk factors (Maximum-Likelihood Chi-square).

Risk Factors		Subpopulation Affected by Ewe Neck (%)	*p*-Value
Sex	Male	28.18 ^b^	<0.005
	Female	26.55 ^a^	
Age	<4 years	25.05 ^a^	<0.005
	4–7 years	28.40 ^c^	
	>7	27.95 ^b^	
Coat Color	Grey	25.26 ^a^	<0.005
	Brown	27.76 ^b^	
	Black	30.80 ^c^	
	Chestnut	32.37 ^d^	
Geographical area	Spain	26.52 ^d^	<0.005
	North America	32.03 ^c^	
	Central America	29.27 ^e^	
	Europe	22.94 ^a^	
	South America	28.56 ^b^	
	Australia	26.09 ^abc^	
Birth stud size (number of mares)	<5	29.45 ^c^	<0.005
	5–9	28.68 ^c^	
	10–19	27.67 ^c^	
	20–50	25.87 ^a^	
	>50	26.58 ^b^	

Ewe Neck subpopulation (class 1, 2, and 3). Whole population (class 0, 1, 2, and 3). Different superscript letters ^(a, b, c, d, e)^ indicate a significant difference between groups (*p* < 0.05) according to Maximum-Likelihood Chi-square.

**Table 5:** Heritability and genetic correlations ± (s.d.) of Ewe Neck and morphological traits in the three complementary approaches (A, B, and C) in a population of Pura Raza Español horses.

Morphological Traits and Body Indices		Heritability	Genetic Correlations ± S.D. (Ewe Neck with Morphological Traits and Body Indices)		
Approach A	Ewe Neck	0.23 ± 0.01	Approach A	Approach B	Approach C
Approach B		0.24 ± 0.02			
Approach C		0.34 ± 0.03			
	HW	0.77 ± 0.01	−0.15 ± 0.00	−0.17 ± 0.03	−0.10 ± 0.07
	HDPW	0.68 ± 0.01	−0.20 ± 0.03	−0.20 ± 0.04	−0.07 ± 0.05
	HC	0.40 ± 0.02	−0.25 ± 0.04	−0.32 ± 0.05	0.30 ± 0.08
	WH	0.65 ± 0.01	−0.02 ± 0.03	−0.02 ± 0.03	0.08 ± 0.07
	LN	0.33 ± 0.01	−0.11 ± 0.04	−0.10 ± 0.04	0.04 ± 0.07
	LS	0.49 ± 0.02	−0.04 ± 0.03	−0.17 ± 0.03	0.49 ± 0.05
	WC	0.33 ± 0.01	0.00 ± 0.03	0.12 ± 0.04	−0.38 ± 0.08
	SIL	0.55 ± 0.02	0.02 ± 0.03	0.06 ± 0.03	−0.05 ± 0.07
	Lba	0.48 ± 0.01	−0.25 ± 0.01	−0.36 ± 0.03	0.47 ± 0.05
	LL	0.49 ± 0.01	0.18 ± 0.03	0.09 ± 0.03	0.48 ± 0.05
	DSD	0.37 ± 0.01	0.04 ± 0.04	0.17 ± 0.04	−0.46 ±0.07
	PT	0.57 ± 0.01	−0.14 ± 0.03	−0.15 ± 0.03	0.10 ± 0.06
	AS	0.31 ± 0.02	−0.10 ± 0.04	−0.10 ± 0.04	0.07 ± 0.06
	MD	0.32 ± 0.01	−0.12 ± 0.04	−0.14 ± 0.04	−0.11 ± 0.08
	HNJ	0.26 ± 0.01	−0.15 ± 0.04	−0.30 ± 0.04	0.57 ± 0.05
	NBJ	0.13 ± 0.01	−0.38 ± 0.04	−0.38 ± 0.04	−0.38 ± 0.04
	Prl	0.46 ± 0.01	0.04 ± 0.03	0.07 ± 0.04	−0.29 ± 0.07
	HI	0.38 ± 0.02	−0.22 ± 0.03	−0.19 ± 0.03	−0.34 ± 0.06
	TI	0.50 ± 0.02	−0.25 ± 0.03	−0.27 ± 0.03	−0.07 ± 0.08
	BI	0.27 ± 0.01	−0.15 ± 0.04	−0.19 ± 0.04	0.25 ± 0.07

Height at withers (HW); height at decline point of withers (HDPW); height at chest (HC); width of head (WH); length of neck (LN); length of shoulder (LS); width of chest (WC); scapulo-isquial length (SIL); length of back (LBa); length of loin (LL); dorso-sternum diameter (DSD); perimeter of thorax (PT); angle of shoulder (AS); muscle development (MD); head neck junction (HNJ); bottom neck-body junction (NBJ); proportionality index (Prl); head index (HI); thoracic index (TI); body index (BI); standard deviations (S.D.).
